# Scandinavian trial of uncomplicated aortic dissection therapy: study protocol for a randomized controlled trial

**DOI:** 10.1186/s13063-023-07255-7

**Published:** 2023-03-23

**Authors:** Claudina Rudolph, Beate Rikken Lindberg, Timothy Resch, Kevin Mani, Patrick Björkman, Elín Hanna Laxdal, Henrik Støvring, Henriette Margrethe Beck, Gunnar Eriksson, Jacob Budtz-Lilly

**Affiliations:** 1 Division of Vascular Surgery, Department of Cardiovascular Surgery, Aarhus University Hospital, Aarhus, Denmark; 2 Department of Cardiothoracic Surgery, Oslo University Hospital, Rikshospitalet, Oslo Norway; 3 Department of Vascular Surgery, Rigshospitalet, Copenhagen, Denmark; 4 Department of Clinical Medicine, Faculty of Health and Medical Sciences, University of Copenhagen, Copenhagen, Denmark; 5 Section of Vascular Surgery, Department of Surgical Sciences, University of Uppsala, Uppsala, Sweden; 6 Department of Vascular Surgery, Abdominal Center, Helsinki University Hospital, Helsinki, Finland; 7 Department of Vascular Surgery, Landspitali University Hospital, Reykjavik, Iceland; 8 Department of Public Health, Aarhus University, Aarhus, Denmark

**Keywords:** Aneurysm, Dissecting, TEVAR, Type B, Stent graft, Uncomplicated, Aortic dissection, TEVAR

## Abstract

**Background:**

Contemporary management of uncomplicated type B aortic dissections (uTBAD) is based on the acuity and various morphological features. Medical therapy is mandatory, while the risks of early thoracic endovascular aortic repair (TEVAR) are balanced against the potential for rupture, complex surgery, and death. Improved aortic morphology following TEVAR is documented, but evidence for improved overall survival is lacking. The costs and impact on quality of life are also needed.

**Methods:**

The trial is a randomized, open-label, superiority clinical trial with parallel assignment of subjects at 23 clinical sites in Denmark, Norway, Sweden, Finland, and Iceland. Eligibility includes patients aged ≥ 18 with uTBAD of < 4 weeks duration. Recruited subjects will be randomized to either standard medical therapy (SMT) or SMT + TEVAR, where TEVAR must be performed between 2–12 weeks from the onset of symptoms.

**Discussion:**

This trial will evaluate the primary question of whether early TEVAR improves survival at 5 years among uTBAD patients. Moreover, the costs and the impact on quality of life should provide sorely needed data on other factors that play a role in treatment strategy decisions. The common Nordic healthcare model, with inclusion of all aortic centers, provides a favorable setting for carrying out this trial, while the robust healthcare registries ensure data validity.

**Trial registration:**

ClinicalTrials.gov NCT05215587. Registered on January 31, 2022.

## Administrative information

Note: the numbers in curly brackets in this protocol refer to SPIRIT checklist item numbers. The order of the items has been modified to group similar items (see http://www.equator-network.org/reporting-guidelines/spirit-2013-statement-defining-standard-protocol-items-for-clinical-trials/).

**Table:** 

Title {1}	Scandinavian trial of uncomplicated aortic dissection therapy: study protocol for a randomized controlled trial
Trial registration {2a and 2b}.	ClinicalTrials.gov: NCT05215587
Protocol version {3}	01 February, 2023, Version 11.0
Funding {4}	Vetenskapsrådet (Swedish Research Council) Hjärt-Lungfonden (Swedish Heart and Lung Foundation) Both of these funding bodies are entirely independent and have played no role in the design of the protocol nor the planned collection, analysis, and interpretation of the data.
Author details {5a}	^1^ Division of Vascular Surgery Department of Cardiovascular Surgery Aarhus University Hospital Aarhus, Denmark ^2^ Department of Cardiothoracic Surgery Oslo University Hospital, Rikshospitalet Oslo, Norway ^3^Department of Vascular Surgery Rigshospitalet, Copenhagen, Denmark Department of Clinical Medicine, Faculty of Health and Medical Sciences, University of Copenhagen Copenhagen, Denmark ^4^ Section of Vascular Surgery Department of Surgical Sciences University of Uppsala Uppsala, Sweden ^5^ Department of Vascular Surgery Helsinki University Hospital, Abdominal Center Helsinki, Finland ^6^ Department of Vascular Surgery Landspitali University Hospital Reykjavik, Iceland
Name and contact information for the trial sponsor {5b}	Clinical investigator driven trial, sponsored by the primary investigator, Jacob Budtz-Lilly.
Role of sponsor {5c}	Not applicable.

## Introduction

### Background and rationale {6a}

Theincidence of a Stanford type-B thoracic aortic dissection (TBAD) is estimated at 3.9–6.0 per 100,000 person-years, although this may be an underestimate [1–3]. These account for approximately 30–40% of all types of aortic dissection [4]. The diagnosis of TBAD is further classified with respect to timing: acute, ≤ 14 days, subacute, 15–90 days, and chronic, > 90 days. Approximately 40–50% of TBADs are considered complicated and defined by the presence of one or more of the following: rupture and/or hypotension/shock, organ malperfusion, rapid aortic expansion, paraplegia/paraparesis, peri-aortic hematoma, or intractable pain or hypertension [2, 5]. The definition of intractable is somewhat vague in the literature, yet the guidelines from the American Society for Vascular Surgery suggest a duration of > 12 h despite medical therapy [6]. In the absence of these complications, the dissection is considered uncomplicated. In-hospital survival for these patients has been reported as approximately 90% [7].

Open surgery has previously played a role in the treatment of TBAD patients, but its dismal outcomes, particularly when compared to medical treatment, have led to changes in strategy [8–10]. Contemporary TBAD management is dependent upon the above-mentioned factors, i.e., complicated or uncomplicated, acute or chronic, as well as accompanying comorbidities. An underlying and universal component for all these patients is standard medical therapy, which includes antihypertensive therapy, typically β-blockers, in order to mitigate aortic wall stress and false lumen pressures, as well as pain relief [11]. Furthermore, lifestyle improvements and cardiovascular risk profile modification are recommended.

The introduction of thoracic endovascular aortic repair (TEVAR) in 1994 radically changed the treatment of TBAD, and TEVAR is now the recommended therapy for complicated TBADs, thoracic aortic aneurysms, and traumatic thoracic transections, among others [12, 13]. To date, the use of TEVAR in the treatment of uncomplicated TBAD is uncertain, if not controversial. Several analyses have found that TEVAR confers improved aortic remodeling and possibly survival, albeit with the implied and inherent procedural risks of intervention, including paraplegia, retrograde dissection, and death [14, 15].

There are two relevant randomized clinical trials (RCTs), addressing the issue of early TEVAR among TBAD patients. The Acute Dissection: Stent graft OR Best medical therapy (ADSORB) trial, notably underpowered, randomized a total of 61 patients from 17 European centers with acute uTBAD [16]. There were no aortic ruptures at 1 year in either arm of the trial, while TEVAR was associated with improved thrombosis of the false lumen and reduction of its lumen. The Investigaton of Stent Grafts in Aortic Dissection (INSTEAD) trial included 140 patients in the subacute phase [17]. The overall survival at two years was statistically equivalent, 95.6% in the OMT (optimal medical therapy) group and 88.9% in the TEVAR plus OMT group. The long-term results from the extended INSTEAD-XL found a non-significant absolute reduction in all-cause mortality of 8.2% at 5 years for those patients who underwent TEVAR [18]. The authors performed an additional Landmark analysis, thus focusing only on outcomes from years two to five, and identified an absolute mortality reduction of 16.9%.

The conclusions from the retrospective and above-mentioned RCTs have not been persuasive enough for the European Society of Vascular Surgery to render a higher recommendation than “TEVAR may be selectively considered” for those patients presenting with uncomplicated type B aortic dissections [2]. This is furthermore echoed by a recent international survey regarding the preferred treatment of uTBAD, in which 54.8% of respondents answered that they do not routinely use TEVAR, as opposed to 37.4% who prefer this strategy. More importantly, 88.6% of respondents agreed that equipoise was present and that an RCT was needed [19].

Notwithstanding the clinical implications of various treatment algorithms, there are two further relevant aspects regarding the treatment of uTBAD patients that must be considered. First, the economic ramifications of potential interventions, readmissions, reinterventions, and rehabilitation are complex. A recent Canadian study demonstrated that the median and total yearly costs of treating aortic dissection have increased beyond the rate of inflation, while rehabilitation constitutes a significant portion of these costs [20]. Second, and somewhat coupled to the first, is the quality of life of these patients. Although evidence is limited, patients surviving a dissection have reported poorer levels of mental health and sexual function [21]. These two issues must be accounted for in any future societal appraisals of the evidence and evaluations of the costs and benefits.

### Objectives {7}

The primary objective is to compare the overall survival at 5 years between subjects treated with standard medical therapy (SMT) or SMT + subacute TEVAR. The secondary objectives include the comparison of aortic-related mortality, neurological injury, aortic intervention, readmissions, reinterventions, quality-of-life (QoL), costs, and 10-year survival.

### Trial design {8}

The trial is a randomized, open-label, superiority clinical trial with parallel assignment of subjects in multiple sites in Denmark, Sweden, Norway, Iceland, and Finland. Recruited subjects will be randomized to either SMT exclusively or TEVAR + SMT.

## Methods: participants, interventions, and outcomes

### Study setting {9}

The study encompasses all of the 23 academic and tertiary aortic centers in the five above-given Nordic countries. These sites represent the major referral centers for medical and surgical treatment of aortic pathology. An updated list on participating sites, recruitment, and contact information is found on the trial [22].

### Eligibility criteria {10}

#### Inclusion criteria

All subjects, aged 18 or greater at the time of informed consent signature, admitted or referred to the participating cardiovascular Sites with an uTBAD of less than four weeks duration.

#### Exclusion criteria

- Subjects with no signed informed consent.
- Subjects presenting with a *complicated* type B aortic dissection according to the above definition.
- Subjects previously treated in their descending aorta, either open surgery or TEVAR.
- Subjects with pre-existing thoracoabdominal aortic aneurysm.
- Subjects with traumatic aortic dissections.
- Subjects with an established connective tissue disease at the time of randomization, including but not limited to Marfans and Loeys-Dietz syndrome.
- Subjects with a clinically estimated life expectancy < 2 years.
- Subjects with dementia.
- Pregnant or nursing subjects.
- Subjects with current sepsis.
- Subjects currently participating in other clinical interventional trials.

### Who will take informed consent? {26a}

The process of informed consent will be carried out by an approved primary investigator from any of the participating sites. Consent procedures follow local and national regulatory guidelines. A copy of the informed consent document will be given to the subject for their records.

### Additional consent provisions for collection and use of participant data and biological specimens {26b}

No additional or ancillary data will be obtained and, thus, no further consent will be obtained.

## Interventions

### Explanation for the choice of comparators {6b}

Despite evidence from retrospective and descriptive studies suggesting long-term benefits for early TEVAR intervention among uTBAD subjects, the underlying unanswered question is whether TEVAR confers a benefit of survival. The two previous RCTs, mentioned above, were underpowered to address this issue. Despite the potential theoretical and procedural advantages of various composite endpoints, it was determined that a trial based on a clearly expressed question with a binary outcome will have the most clinical impact. Similarly, focus on the albeit interesting, but not essential, endpoint of aortic morphological changes and imaging findings, would complicate the pragmatic design of this trial.

### Intervention description {11a}

#### Standard medical therapy (SMT)

Contemporary standard medical therapy for TBAD consists of antihypertensive agents and pain relief. The choice of the specific agents will be left to the discretion of the individual treatment sites/surgical team, based on the individual subject’s prior and current therapy and tolerance to various medical regimens. While the goal is to reduce the systolic blood pressure to between 100 and 120 mm Hg and the pulse rate below 60 beats/minute in the acute phase, the advocated first-line therapy consists of intravenous β-blockade, with calcium channel antagonists and/or renin-angiotensin inhibitors as alternatives. Pain relief is furthermore critical in order to mitigate activation of the sympathetic nervous system and resultant tachycardia and blood pressure elevation. Anxiolytic medication may also be used in this role.

Long-term SMT is essential and, although not evaluated in any clinical trials, the target blood pressure is 120/80 mmHg [23]. All subjects will be equipped with a home blood pressure apparatus in order to measure and record their values. As detailed below, these measurements will be recorded in the electronic database for all subjects at follow-up consultations.

Clearly, medical therapy for aortic dissection is a complex and unresolved research topic in and of itself, and individual-specific therapy can only be supported by guidelines from the European Society of Vascular Surgery and the European Society of Cardiology. Consideration in the trial was given to the connotations of “best” or “optimal” medical therapy, as well as kindred RCT protocols, e.g., Asymptomatic Carotid Surgery Trial-1 (ACST-1) [24], and the ramifications of these definitions vis-à-vis endpoint determination. Because of the recognized local differences in medical therapy and the interest in maintaining the pragmatic nature of this trial, it was determined that the terminology of “standard medical therapy” is most appropriate.

To that end, all sites, investigators, and subjects will be informed of the blood pressure target-oriented nature of this treatment and the following recommendations from the European Society of Vascular Surgery: Initial therapy consists of β-blockers. In subjects who do not respond to β-blockers or who do not tolerate the drug, calcium channel antagonists and/or renin-angiotensin inhibitors can be used as alternatives [2]. In addition to these recommendations for hypertension, efforts should be made to alter and improve lifestyle and cardiovascular risk profiles, including smoking cessation, weight control, and potential treatment of other comorbidities such as diabetes mellitus and ischemic heart disease.

#### Thoracic endovascular aortic repair (TEVAR)

Subjects randomized to TEVAR therapy will undergo placement of an endovascular stent graft in the descending thoracic aorta. The selection of the stent graft is left to the discretion of the treating physicians. While the implicit goal of TEVAR in dissection treatment is to treat the primary tear, certain adjunct proximal and/or distal procedures are often required, e.g., coverage of the left subclavian artery with or without a supplementary left subclavian artery revascularization, e.g., left carotid artery-to-left subclavian artery bypass/transposition or fenestration to left subclavian artery. Any or all adjunct procedures deemed necessary or beneficial by the treating physicians and subjects are allowable under the allocation to the TEVAR subject cohort, as this reflects real-world considerations and the question at hand based on analysis of an intention-to-treat. This includes distal or proximal aortic sealing, as well as Provisional Extension To Induce Complete Attachment (PETTICOAT) or Stent-Assisted Balloon-Induced Intimal Disruption and Relamination in Aortic Dissection Repair (STABILISE) [25, 26].

### Criteria for discontinuing or modifying allocated interventions {11b}

All subjects receive SMT, regardless of the allocated arm of the trial, while the interventional arm will undergo TEVAR treatment within 12 weeks from the onset of symptoms. Some SMT subjects may require surgery, including TEVAR, and the indications for this are up to the discretion of the treating medical and surgical multi-disciplinary team. Indications include, but are not limited to: rupture, rapid aortic expansion, total aortic diameter ≥ 6 cm, pain, refractory hypertension, or malperfused organ(s). Both the procedure and indication will be registered. Note that any intervention predicated on the original aortic pathology is considered as one of the secondary outcomes, i.e., aortic intervention.

### Strategies to improve adherence to interventions {11c}

The premise of this trial is based on both pragmatism and on an intention to treat. Any screened subject with an uTBAD will therefore be considered for recruitment. It is important to underscore, however, that discretion for recruitment is relegated to the sites and investigators, as they must consider the clinical ramifications and the safety of the subject. Significant efforts have been made in informing centers that the limited exclusion criteria should allow for increased enrolment and should discourage arbitrary exclusion based on erroneous protocol interpretations.

### Relevant concomitant care permitted or prohibited during the trial {11d}

As medical therapy is a critical component of both arms of the trial, there are no anticipated egregious therapies that require explicit prohibition. Furthermore, the TEVAR procedure, as defined above, is given broad allowances for adjunct procedures, both planned and ad hoc. All hospital readmissions will also be registered.

### Provisions for post-trial care {30}

All patients enrolled in this trial will be treated and followed in the public healthcare system. There are no private institutions or practitioners involved. Furthermore, all patients will have a documented diagnosis of uTBAD, i.e., there are no otherwise healthy control patients who undergo an experimental treatment. Finally, TEVAR is a recognized and CE (Conformité Européenne) -marked treatment and should in no manner be considered a form of alternative treatment. The right for compensation will thus be considered under the standard avenues and regulations for patient compensation, as covered by each of the national healthcare regulations among the five participating countries.

### Outcomes {12}

The endpoints will be collected from the electronic database and correlated, where relevant, with the individual national board of health registries. These definitions are in accordance with the guidelines from the European Society of Vascular Surgery and the reporting standards document from the American Society for Vascular Surgery [2, 6].

#### Primary outcome

All-cause mortality.

#### Secondary outcomes

##### Aortic-related mortality

Death as a result of aortic rupture or organ malperfusion, or death due to aortic intervention.

##### Aortic intervention

Any open surgical or endovascular intervention performed in any anatomical location, performed for the following indications, which are related to the aortic pathology: aneurysmal degeneration, visceral ischemia, lower extremity ischemia, rupture, or any of the criteria listed above under the definition of a complicated TBAD [2, 27]. Both the timing and indication for the aortic intervention should be recorded. Importantly, the decision for intervention is at the discretion of the treating physician and medical team.

##### Neurological injury

These are divided into two categories: cerebrovascular accidents (CVA) and spinal cord ischemia (SCI). CVAs are defined according to the Society for Vascular Surgery reporting standards and classified as any central neurological complication, ischemic and hemorrhagic. For this project, the modified Rankin scale will be used for classifying stroke severity (Table 1) [28]. Spinal cord ischemia is defined as either ischemic or hemorrhagic resulting in paraparesis or paraplegia. The modified Tarlov scoring scale will be used for the grading of any spinal cord injuries (Table 2) [29]. It is recommended, but not mandatory, that an independent neurologist be consulted for this purpose.

**Table 1:** Modified Rankin scale for stroke severity [28]

The scale runs from 0 to 6, running from perfect health without symptoms to death	
0	No symptoms
1	No significant disability. Able to carry out all usual activities, despite some symptoms
2	Slight disability. Able to look after own affairs without assistance, but unable to carry out all previous activities
3	Moderate disability. Requires some help, but able to walk unassisted
4	Moderately severe disability. Unable to attend to own bodily needs without assistance, and unable to walk unassisted
5	Severe disability. Requires constant nursing care and attention, bedridden, incontinent
6	Dead

**Table 2:** Modified Tarlov scoring scale for spinal cord injury [29]

Scale	Motor function	Deficit
0	No lower extremity movement	Paraplegia
1	Lower extremity motion without gravity	Paraplegia
2	Lower extremity motion against gravity	Paraplegia
3	Able to stand with assistance	Paraparesis
4	Able to walk with assistance	Paraparesis
5	Normal	Normal

##### Reintervention

Any open or endovascular intervention after the original TEVAR procedure that was related to the dissection. These should be categorized as either planned reintervention, e.g., a staged procedure, or unplanned, which indicates a complication from the original procedure, a failure of the device, or progression of disease.

##### Quality of life

The quality of life will be assessed with the three following self-assessment forms:

1. The EuroQOL-5D-5L instrument from the EuroQol Group, comprised of five dimensions with five levels of scoring that can be combined into a five-digit number of description [30].
2. The Hospital and Anxiety Depression Score (HADS) [31].
3. The 12-Item Short-Form (12-SF) Health Survey [32].

#### Economic evaluation

The economic evaluation will be performed from a payer/healthcare point of view, including resource use associated with healthcare, intervention, and medication, whereas broader potential consequences for society, i.e., effects on productivity, will not be included. During the course of the trial, the accumulated costs will be measured per treatment arm from the participating hospital´s administrative/controlling/billing systems. As far as possible, the following resource use items will be included and captured as accumulated costs from the hospital’s cost-per-subject system on all outpatient and inpatient visits:

- Costs for healthcare staff
- Subject-specific costs for primary and secondary endovascular and surgical procedures postoperative care unit costs
- Costs of drugs during surgery and postoperative care
- Costs of anesthetic procedures and blood transfusions
- Additional diagnostic procedures from the radiology and clinical physiology departments and from clinical chemistry.

The costs for healthcare staff will comprise the full wage costs, including costs for social security. Costs for each endovascular and surgical procedure will be retrieved individually, and, as far as possible, be based on the price per minute according to the hospital’s cost-per-subject systems.

Changes in health status will be assessed in terms of quality-adjusted life-years (QALYs), which combine the time spent in a specific health state with the corresponding.self-assessed health-related quality of life (HRQoL), as derived from the EuroQOL EQ-5D-5L questionnaire. Time is measured in years and the HRQoL is measured on an index scale ranging from 0 (equivalent to being dead) to 1 (best possible health state). The total number of QALYs will be calculated by multiplying the HRQoL index score (QALY weight) by the time spent in each health state. Group differences in total costs will be calculated and divided by the difference in QALYs in the interval from baseline until the end of the study, and the incremental cost-effectiveness ratio will be calculated as follows:

$$(\mathrm{CostTEVAR }-\mathrm{ CostSMT})/(\mathrm{QALYsTEVAR }-\mathrm{ QALYsSMT}) =\mathrm{ \Delta Cost}/\mathrm{\Delta QALY}$$

#### Rationale for objectives and endpoint selection

Despite evidence from retrospective and descriptive studies suggesting long-term benefits for early TEVAR intervention among uTBAD subjects, the underlying unanswered question is whether TEVAR confers a benefit of survival. The two previous RCTs, mentioned above, were underpowered to address this issue. Despite the potential theoretical and procedural advantages of various composite endpoints, it was determined that a trial based on a clearly expressed question with a binary outcome will have the most clinical impact. Similarly, focus on the albeit interesting, but not essential, endpoint of aortic morphological changes and imaging findings, would complicate the pragmatic design of this trial.

### Participant timeline {13}

**Fig. 1:**
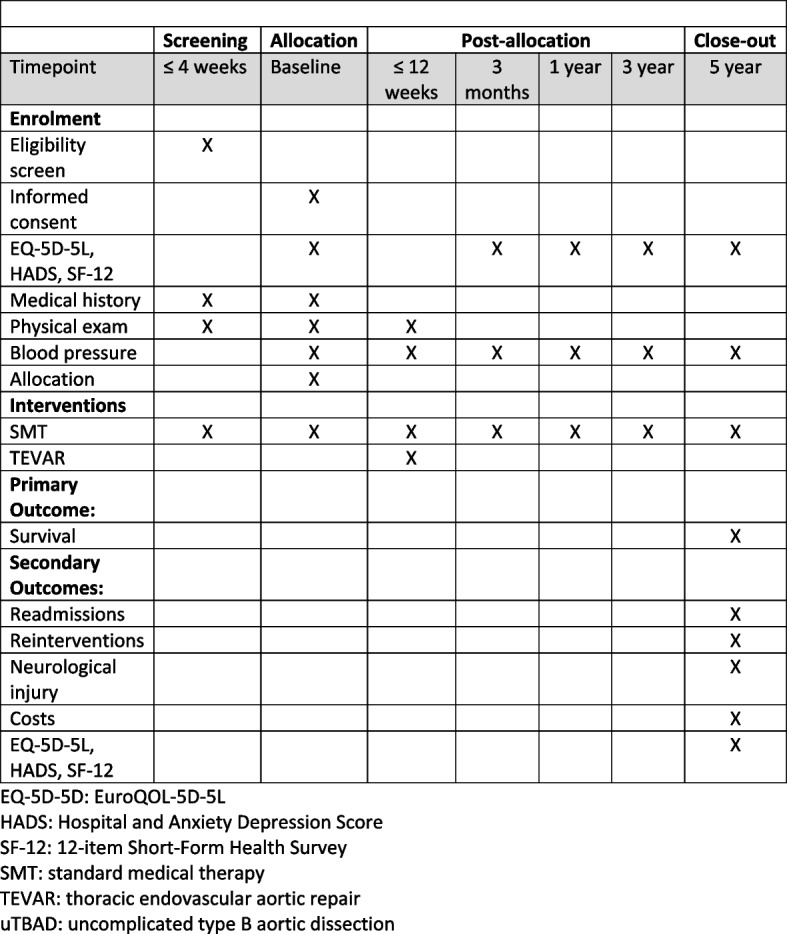
SPIRIT figure. EQ-5D-5D, EuroQOL-5D-5L; HADS, Hospital and Anxiety Depression Score; SF-12, 12-item Short-Form Health Survey; SMT, standard medical therapy; TEVAR, thoracic endovascular aortic repair; uTBAD, uncomplicated type B aortic dissection

The schedule of enrolment, interventions, and assessments timeline is depicted schematically in Fig. 1.

### Sample size {14}

The literature supports an overall estimated 5-year survival for TBAD subjects of approximately 80%, i.e., a 5-year mortality of 20% [33]. The above-mentioned INSTEAD-XL clinical trial identified a reduction in 5-year mortality from 19.3 to 11.1% for those who were randomized to TEVAR, corresponding to a hazard ratio of 0.52 [18]. The estimate was not statistically significant, possibly due to a low sample size.

The primary analysis will be based on the intention to treat principle using the full analysis set and all-cause mortality events as confirmed by the local investigator. The primary objective of the study is to determine the superiority of TEVAR versus SMT in reducing the incidence of all-cause mortality. Assuming a true hazard ratio of 0.52 between TEVAR and SMT, using a two-sided alpha of 5%, 80 subjects with primary endpoint events will provide a statistical power of 80% for the test of all-cause mortality between treatment arms, based on an overall 1:1 allocation between TEVAR and SMT and analyzed with a log-rank test. All Scandinavian countries have registries of vital statistics with a high reliability and almost complete follow-up. Consequently, loss-to-follow-up is expected to be negligible, except for migration, and thus a conservative estimate of loss-to-follow-up is given as 3%.

The study is event-driven. With an estimated annual event rate of 4% for the primary endpoint in the control group and a withdrawal probability of 3%, approximately 550 subjects are estimated to provide the required number of primary events.

In summary, the parameters used for the power calculation are as follows:

$$\begin{array}{c}\mathrm{Log}-\mathrm{rank}\;\mathrm{test}\;\mathrm{for}\;\mathrm{comparison}\;\mathrm{of}\;\mathrm{two}\;\mathrm{groups}\\\mathrm{Two}-\mathrm{sided}\;\mathrm{significance}\;\mathrm{level}\;(\mathrm{alpha})=0.05\;(5\%)\\\mathrm{Power}\;(1-\mathrm{Beta})=0.80\;(80\%)\\\mathrm{Hazard}\;\mathrm{ratio}=0.52\\\mathrm{Withdrawal}\;\mathrm{probability}=0.03\;(3\%)\\\mathrm{Inflation}\;\mathrm{factor}\;\mathrm{due}\;\mathrm{to}\;\mathrm{single}\;\mathrm{interim}\;\mathrm{analysis}\;(\mathrm{see}\;\mathrm{below})=1.0071\\\mathrm{Estimated}\;\mathrm{number}\;\mathrm{of}\;\mathrm{events},\;\mathrm i.\mathrm e.\;\mathrm{deaths}=80\\\mathrm{Estimated}\;\mathrm{total}\;\mathrm{sample}\;\mathrm{size}=550\times1.0071=554\\\mathrm{Estimated}\;\mathrm{number}\;\mathrm{in}\;\mathrm{each}\;\mathrm{arm}=225\times1.0071=277\end{array}$$

### Recruitment {15}

With the broad and full participation of all aortic centers that treat uTBAD patients in Nordic countries, the potential for recruitment of subjects is favorable. Involvement of representatives from all five countries in the Trial Steering Committee (TSC) also encourages shared ownership, as has the inclusion of experts from multiple centers in the various discussions regarding the design of the trial.

## Assignment of interventions: allocation

### Sequence generation {16a}

The process of randomization will be carried out by the same third-party electronic dataset system, Research Electronic Data Capture (REDCap), registered at Aarhus University, with 24-h access. Because of the multi-site nature of the trial, randomization will be stratified by the number of clinical sites.

### Concealment mechanism {16b}

As indicated above, randomization or allocation will be performed by REDCap. This electronic dataset is password protected, thus ensuring the concealment of the randomization sequence.

### Implementation {16c}

As noted above, the randomization to either arm will be allocated via REDCap, a process carried out as soon as the acquisition of informed consent is provided in the electronic database.

## Assignment of interventions: blinding

### Who will be blinded {17a}

Due to the nature of one interventional arm of the trial, it is impossible to blind both subjects and investigators to the assignment of treatment. Blinding will be enforced, however, for the analysis of data.

### Procedure for unblinding if needed {17b}

The design is open-label, while data analysis is blinded, so unblinding is not deemed necessary and will not occur.

## Data collection and management

### Plans for assessment and collection of outcomes {18a}

Site initiation prior to approval for subject enrolment includes instructions on data collection and registration in REDCap. Each site has a primary investigator, who is responsible for either registering the data or delegating this responsibility to an approved research assistant. A Trial Management File is kept at each participating site, in addition to a Trial Management File for the entire study group.

### Plans to promote participant retention and complete follow-up {18b}

The typical course of uTBAD requires long, and typically, life-long surveillance. The trial is deliberately designed to mirror the typical follow-up protocol for these patients, thus ensuring participant retention.

### Data management {19}

Site initiation includes instruction in data entry and security, of which the site primary investigator is responsible. The Data and Safety Monitoring Committee (DSMC) will further perform evaluation of the database for data completion and biannual validation of recorded data. The instructions, delegation, and responsibility for these tasks are recorded in each Trial Managers Files and Data Source Manuals.

### Confidentiality {27}

No personal identification numbers are recorded in the electronic database, but rather linked to a separate and secure identification list at each site. Part of each national ethical application was an approval of the processes to ensure confidentiality. Each patient receives oral and written information regarding data protection and their rights as a participant in the trial.

### Plans for collection, laboratory evaluation, and storage of biological specimens for genetic or molecular analysis in this trial/future use {33}

No biological material will be collected in this trial.

## Statistical methods

### Statistical methods for primary and secondary outcomes {20a}

Both an intention-to-treat and a per-protocol analysis will be performed. As mentioned, in the primary analysis, survival rates will be compared using the log-rank test. Before analysis, log–log-survival versus time plots will be used to visually assess the assumption of proportional hazards supported by statistical tests based on the inclusion of a time-varying covariate for the treatment effect. In the case of crossing survival curves, the overall log-rank test will be reported together with effect estimates in-between crossings. If repeated crossings occur, this will in itself suggest that the intervention does not result in superior outcomes for the subjects, which will then be reported.

### Interim analyses {21b}

Given the long duration of the study and the potential for achieving sufficient evidence prior to the end of follow-up, as well as the potential for safety issues, a single interim analysis is planned. The interim analysis will be undertaken when approximately half of the total events have occurred, i.e., 40 events. This will use the O’Brien-Fleming boundary with a two-sided significance level of 0.0052 in conjunction with the log-rank test. This virtually preserves the overall type I error rate (4.8% vs. 5.0%), and thus the final analysis at the end of the follow-up will be conducted with the conventional significance level of 0.05 [34].

### Methods for additional analyses (e.g., subgroup analyses) {20b}

To explore treatment effect heterogeneity in sub-groups, e.g., female patients, Cox Proportional Hazards (Cox PH) regression will be used.

### Methods in analysis to handle protocol non-adherence and any statistical methods to handle missing data {20c}

Both an intention-to-treat and per-protocol analysis will be performed. The pragmatic nature of the trial favors recruitment, but at the cost of potential risk of deviation from the protocol. Protocol deviations will be recorded and followed by the DSMC, as well as the TSC for any potential impact on the scientific soundness of the study.

The primary outcome of survival will be cross-validated with each of the respective national registries in order to minimize missing outcome data. Sensitivity analyses will be performed to evaluate the possible impact from missing baseline or follow-up data. Finally, inverse probability weighting will be considered for secondary outcomes and subgroup analyses.

### Plans to give access to the full protocol, participant-level data and statistical code {31c}

The full protocol is available upon request and is also available as a PDF on the trial website [22].

## Oversight and monitoring

### Composition of the coordinating center and trial steering committee {5d}

The Trial Steering Committee (TSC) consists of nine members, with a minimum of one representative from each of the five Nordic countries. In addition, there is one statistician and two laypersons, both former uTBAD patients, and one of whom is now the chairperson for the Scandinavian Aorta Dissection patient group. Each national representative is responsible for internal coordination, while the primary international coordinating center is based in Aarhus, Denmark. The coordinating center is led by JBL and two clinical research nurses.

### Composition of the data monitoring committee, its role and reporting structure {21a}

The DSMC is an independent, external expert group with the explicit purpose of protecting and serving the trial participants and to advise the TSC, so as to protect the validity and credibility of the trial. There are three voting members, in addition to an independent and non-voting statistician. None of these members have competing interests. According to their charter, they will meet both prior to recruitment initiation, at least once within six months following initiation, and at least twice annually in the form of either closed or open sessions. Minutes from all meetings will be archived. Each meeting will result in a recommendation to the TSC to continue, terminate, or modify the trial.

### Adverse event reporting and harms {22}

Each participating center and country have specific protocols regarding the reporting of serious adverse events (SAEs) to their relevant health authorities. These events are also recorded in the REDCap database, which are reviewed by the DSMC biannually.

### Frequency and plans for auditing trial conduct {23}

Through correspondence with the Danish unit of Good Clinical Practice and according to the Medical Device Regulation for a CE-marked medical device (article 74.1), no additional auditing is required beyond the planned and approved MDR ethical approval and DSMC monitoring.

### Plans for communicating important protocol amendments to relevant parties (e.g., trial participants, ethical committees) {25}

Protocol amendments will be reported to each of the relevant national ethical committees, in addition to the DSMC, TSC, and each of the site primary investigators. Submission to a peer-reviewed medical journal is planned following an analysis of the results.

### Dissemination plans {31a}

The protocol has been shared and discussed at multiple vascular surgery conferences. Further information is also available on their website [22].

## Discussion

This project takes on a medically important question that has a direct impact on patients-their survival, their quality of life, and the cost it requires to best treat them. The simple design with a concrete question and binary primary endpoint is a strength, and the included evaluations of quality of life and cost are sorely needed. The impact of this study should be immediate.

## Trial status

Protocol Version 11, 10 February 2023. Patient enrolment is anticipated in April 2023 after legal working agreements are completed. Anticipated recruitment period of three years, i.e., recruitment completion as of April 2026.

## Data Availability

The final dataset will remain confidential, and there are no contractual agreements for disclosure to any third parties. Amalgamated data will be available upon agreement with the TSC. A summary of the results will be published on the trial website and sent to interested participants [22].

## Abbreviations

ADSORB: Acute Dissection: Stent graft OR Best medical therapy
CE: Conformité Européenne
CVA: Cerebrovascular accidents
DSMC: Data and safety monitoring committee
HADS: Hospital and Anxiety Depression Score
HRQoL: Health-related quality of life
INSTEAD: Investigation of Stent Grafts in Aortic Dissection
INSTEAD-XL: Investigation of Stent Grafts in Aortic Dissection extended
OMT: Optimal medical therapy
QoL: Quality of life
RCT: Randomized controlled trial
REDCap: Research Electronic Data Capture
SAE: Serious adverse event
SF-12: Short-Form Health Survey
SMT: Standard medical therapy
TBAD: Type B aortic dissection
TEVAR: Thoracic endovascular aortic repair
TSC: Trial steering committee
uTBAD: Uncomplicated type B aortic dissection
